# Bifunctional antibiotic hybrids: A review of clinical candidates

**DOI:** 10.3389/fphar.2023.1158152

**Published:** 2023-06-12

**Authors:** Augustine Jing Jie Koh, Varsha Thombare, Maytham Hussein, Gauri G. Rao, Jian Li, Tony Velkov

**Affiliations:** ^1^ Department of Biochemistry and Pharmacology, School of Biomedical Sciences, Faculty of Medicine, Dentistry and Health Sciences, The University of Melbourne, Parkville, VIP, Australia; ^2^ Monash Biomedicine Discovery Institute, Department of Pharmacology, Monash University, Parkville, VIP, Australia; ^3^ Division of Pharmacotherapy and Experimental Therapeutics, Eshelman School of Pharmacy, University of North Carolina, Chapel Hill, NC, United States; ^4^ Monash Biomedicine Discovery Institute, Department of Microbiology, Monash University, Parkville, VIP, Australia

**Keywords:** antibiotic resistance, drug combinations, hybrid antibiotics, antibiotic pipeline, drug synthesis

## Abstract

Antibiotic resistance is a top threat to human health and a priority across the globe. This problematic issue is accompanied by the decline of new antibiotics in the pipeline over the past 30 years. In this context, an urgent need to develop new strategies to combat antimicrobial resistance is in great demand. Lately, among the possible approaches used to deal with antimicrobial resistance is the covalent ligation of two antibiotic pharmacophores that target the bacterial cells through a dissimilar mode of action into a single hybrid molecule, namely hybrid antibiotics. This strategy exhibits several advantages, including better antibacterial activity, overcoming the existing resistance towards individual antibiotics, and may ultimately delay the onset of bacterial resistance. This review sheds light on the latest development of the dual antibiotic hybrids pipeline, their potential mechanisms of action, and challenges in their use.

## 1 Introduction: the rise of antibiotic resistance

The spread of antibiotic resistance has substantially diminished the efficacies of multiple clinical antibiotics in recent times owing to the emergence and proliferation of multidrug-resistant (MDR) bacterial pathogens (Aminov, 2010; Aslam et al., 2018; Annunziato, 2019). Six pathogenic species, collectively designated ESKAPE (*Enterococcus faecium, Staphyloccocus aureus, Klebsiella pneumonia, Acinetobacter baumannii, Pseudomonas aeruginosa, and Escherichia coli*) were ranked by the World Health Organization (WHO) as high-priority pathogens because of their ability to “escape” multiple antibiotics via resistance mechanisms including enzymatic inactivation, active efflux, and target modification, which can be genetically innate or acquired via horizontal gene transfer (Santajit and Indrawattana, 2016; Mulani et al., 2019; Zhen et al., 2019; de Oliveira et al., 2020; Cardoso et al., 2021). Hence, it is projected the annual mortality rate of antibiotic resistance (10 million) would supersede that of cancer (8.2 million) by 2050 (Dadgostar, 2019; Morris and Cerceo, 2020). This blight is exacerbated by the dearth of the antibiotic pipeline, in which the number of new antibiotics developed has steadily declined over the last 3 decades (Cardoso et al., 2021). With insufficient effective antibiotics, invasive and immunosuppressive treatments would be heavily restricted, and healthcare systems would find themselves extensively overwhelmed by the spread of MDR infections (Aslam et al., 2018; Piret and Boivin, 2021). While prudent stewardship of antibiotics may mitigate resistance, it remains paramount to expand the library of treatment options to compensate for various antibiotics rendered obsolete over the years. One of the means to revive antibiotic efficacy is through antibiotic combinations between synthetic antibiotics and/or last-resort antibiotics.

## 2 Antibiotic combination therapy

**FIGURE 1:**
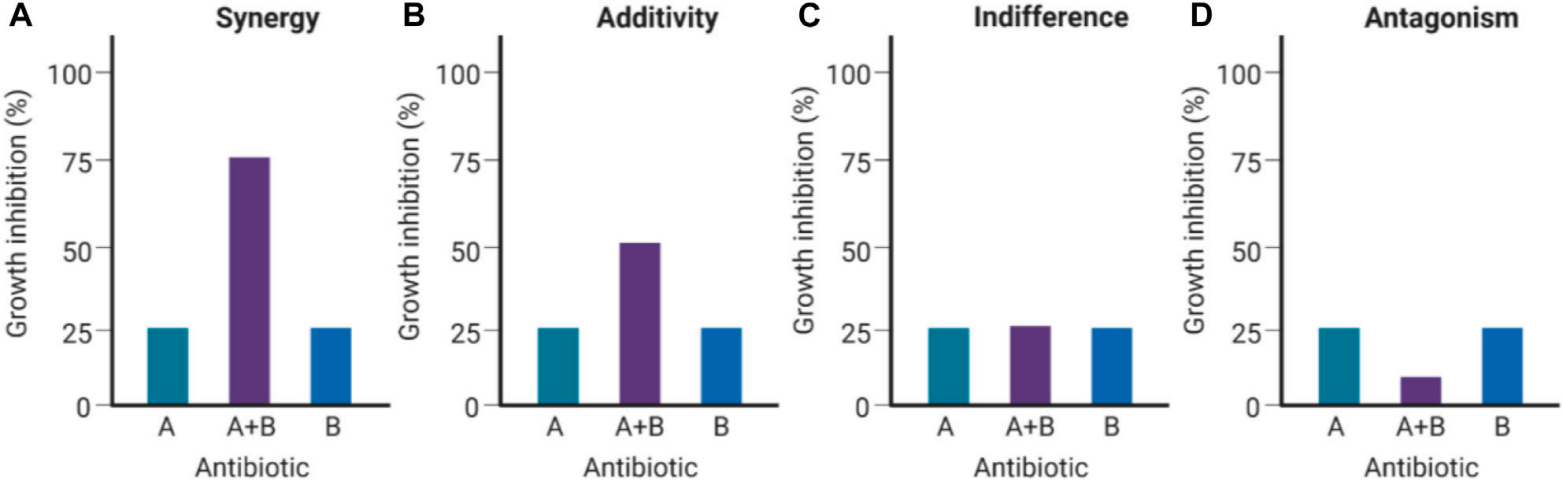
Types of antibacterial interactions for antibiotic combinations (Created with BioRender.com). If the Drug A/B combination is synergistic **(A)**, the combined inhibition of growth (∼75%) would exceed the calculated cumulative antibacterial effect contributed by Drug A (∼25%) and Drug B (∼25%). If the Drug A/B combination is additive **(B)**, the combined inhibition of growth would be equivalent to the calculated cumulative effect (∼50% growth inhibition). If Drugs A and B are indifferent **(C)**, neither of them augments nor subtracts from each other’s efficacies when combined. If Drugs A and B are antagonistic **(D)**, the resulting combined effect results in a considerable decrease in bacterial growth inhibition (Pokrovskaya and Baasov, 2010).

Co-administration of multiple antibiotics has gained increased popularity because the likelihood of pathogens developing resistance against two or more antibiotics concurrently is surmised to be extensively lower than against a single antibiotic administered (Mulani et al., 2019). In addition, any synergistic activity effectuated by the antibiotic combination far exceeds the antibacterial activity of either individual antibiotic in the combination (Figure 1) (Mulani et al., 2019; Theuretzbacher, 2020). Synergy occurs when antibiotics in the cocktail bind two or more targets, or inhibit different vital bacterial metabolic pathways simultaneously, inflicting substantial mortal damage on the bacterial population (Pokrovskaya and Baasov, 2010). However, antibiotic combination therapy incurs significant administrative and therapeutic monitoring costs as *in-vitro* synergy might not necessarily translate to the same effect *in-vivo* owing to a myriad of factors, including non-complementary pharmacodynamic/pharmacokinetic properties, which could have the unintended effect of exacerbating toxicity (Rybap and Mcgrath, 1996; Tamma et al., 2012). An example of antibiotic combinations augmenting toxic side effects is aminoglycoside/cephalosporin combinations, which aggravate nephrotoxicity and thereby, restrict their clinical use (Mannion et al., 1981).

In a cocktail treatment of antibiotics, one of the antibiotics might be short-acting and readily inactivated metabolically or excreted, have different bacterial efflux susceptibilities, or have different tissue distributions compared to the other, where one antibiotic component is scarcely distributed, causing the other component to become vulnerable to resistance development (Domalaon et al., 2018). Differing antibiotic administration routes would also be a cause of inconvenience for patients (Fisher et al., 2020). Furthermore, different antibiotics may be chemically incompatible with one another when mixed. In the case of aminoglycoside/β-lactam co-administration, mixing some aminoglycosides (i.e., gentamicin, tobramycin) with certain *β*-lactams (i.e., carbenicillin, penicillin, and ticarcillin) in the same infusion fluids results in unwanted side reactions, producing inactive complexes (Kradjan and Burger, 1980; Tindula et al., 1983). The extent of inactivation is contingent on the *β*-lactam concentration, contact duration, and temperature (Aminoglycoside antibiotics, 2006).

## 3 Dual hybrid antibiotic therapy

Through scientific ingenuity, the idea of dual hybrid antibiotics materialized. The underlying objective behind ligating two antibiotics together via metabolically stable tethers is to construct a singular heterodimeric entity with a fixed pharmacokinetic profile while retaining the antibacterial mechanisms of the constituent pharmacophores. This could improve on-site targeting, impede bacterial efflux, sterically protect constituent pharmacophores from enzymatic degradation and reduce toxicity when administered *in-vivo* (Pokrovskaya and Baasov, 2010; Theuretzbacher, 2020). One pharmacophore could enhance the bioactivity of the neighbouring pharmacophore to which resistance is developed, potentially expanding its antibacterial spectrum and enabling rapid clearance of pathogens (Pokrovskaya et al., 2009). Moreover, different antibiotic targets (i.e., enzymes, precursor metabolites) can be involved in the same metabolic pathways and are in close physical proximity, which can be readily exploited with hybridized antibiotics through rapid simultaneous binding, readily impairing further development of resistance (Klahn and Brönstrup, 2017). To date, six dual hybrids were reported to have entered clinical trials (Table 1) (World Health Organization, 2021; fiercebiotech, 2018; Surur and Sun, 2021). Commercialization of any of these hybrids would significantly support research interest in developing hybrid antibiotics and provide humanity with a propitious advantage in overcoming the overlooked pandemic of antibiotic resistance. The following section summarizes the synthetic schemes, structural features, pharmacology, and antibacterial mechanisms of dual hybrid antibiotics presently in ongoing clinical evaluation.

**TABLE 1:** Dual hybrid antibiotic clinical candidates.

Candidate	Hybrid class	Molecular weight (Da)	Developer	Mode of action	Current status of clinical development	Reference
TD-1792 (Cefilavancin)	Vancomycin-cephalosporin	1984	Theravance (acquired by R-Pharm)	Binding PBPs and Lipid II	Phase 3, recruiting for cSSSI since 2016	adisinsight, (2022)
TD-1607		2,170			Discontinued in 2021 after two Phase 1 trials, (NCT01791049, NCT01949103, 2013)	Surur and Sun, (2021); Butler and Paterson, (2020); NCT01791049 (2013); NCT01949103 (2013)
TNP-2092	Rifamycin-Fluoroquinolone	1,205	Cumbre Pharmaceuticals (acquired by TenNor Therapeutics)	Binding RNAP and type II topoisomerases	Phase 2 completed in 2020 for ABSSSI (NCT03964493, 2019)	NCT03964493 (2019)
					Phase 1 completed in 2022 for PJI (NCT04294862, 2020)	NCT04294862 (2020)
TNP-2098	Rifamycin-Nitroimidazole	944	TenNor Therapeutics	Binding RNAP and DNA	Phase 2 for *H. pylori*, vaginosis and CDAD ongoing since 2021/2022	Ma et al. (2022)
DNV-3681	Oxazolidinone-Fluoroquinolone	627	Morphochem AG (acquired by Deinove)	Binding ribosomes and type II topoisomerase	Phase 2 (NCT03988855), recruiting for CDAD since 2019	NCT03988855 (2019)
Cadazolid		586	Actelion (Acquired by Johnson & Johnson)		Discontinued in 2018 after two phase 3 trials (NCT01983683, NCT01987895, 2013)	fiercebiotech, (2018); Butler and Paterson, (2020); NCT01987895 (2013); NCT01983683 (1983)

### 3.1 Glycopeptide hybrid candidates

The peptidoglycan cell wall is frequently exploited for antibiotic targeting due to its indispensability in protecting bacteria from osmotic lysis (Nikolaidis et al., 2014; Karas et al., 2020). During the late stage of peptidoglycan synthesis, membrane-bound precursors lipid II shuffle *N*-acetylglucosamine and *N*-acetylmuramic acid to the periplasmic space for peptidoglycan chain elongation. The elongated chains are cross-linked with the main chains, catalyzed by penicillin-binding proteins (PBPs) (Nikolaidis et al., 2014). *β*-Lactams (e.g., penicillin G, methicillin) and glycopeptides (e.g., vancomycin) target this critical stage by binding PBPs and lipid II, respectively (Sauvage and Terrak, 2016). Despite the fact that the discovery of additional *β*-lactam classes and the development of related *β*-lactam derivatives (i.e., 3^rd^/4^th^/5^th^ generation cephalosporins and carbapenems), the expression of stronger classes of *β*-lactamases (i.e., extended-spectrum *β*-lactamases, and carbapenemases), low-affinity PBPs (e.g., PBP2a from methicillin-resistant *S. aureus*, MRSA) and overexpression of efflux pumps continue to substantially diminish *β*-lactam efficacy (Fisher et al., 2005; Worthington and Melander, 2013; Bush and Bradford, 2016; Fisher and Mobashery, 2016). Due to widespread *β*-lactam-resistance, glycopeptides have been administered as last resort alternatives (McCormick et al., 1955; Bush and Bradford, 2016; Cong et al., 2020).

**FIGURE 2:**
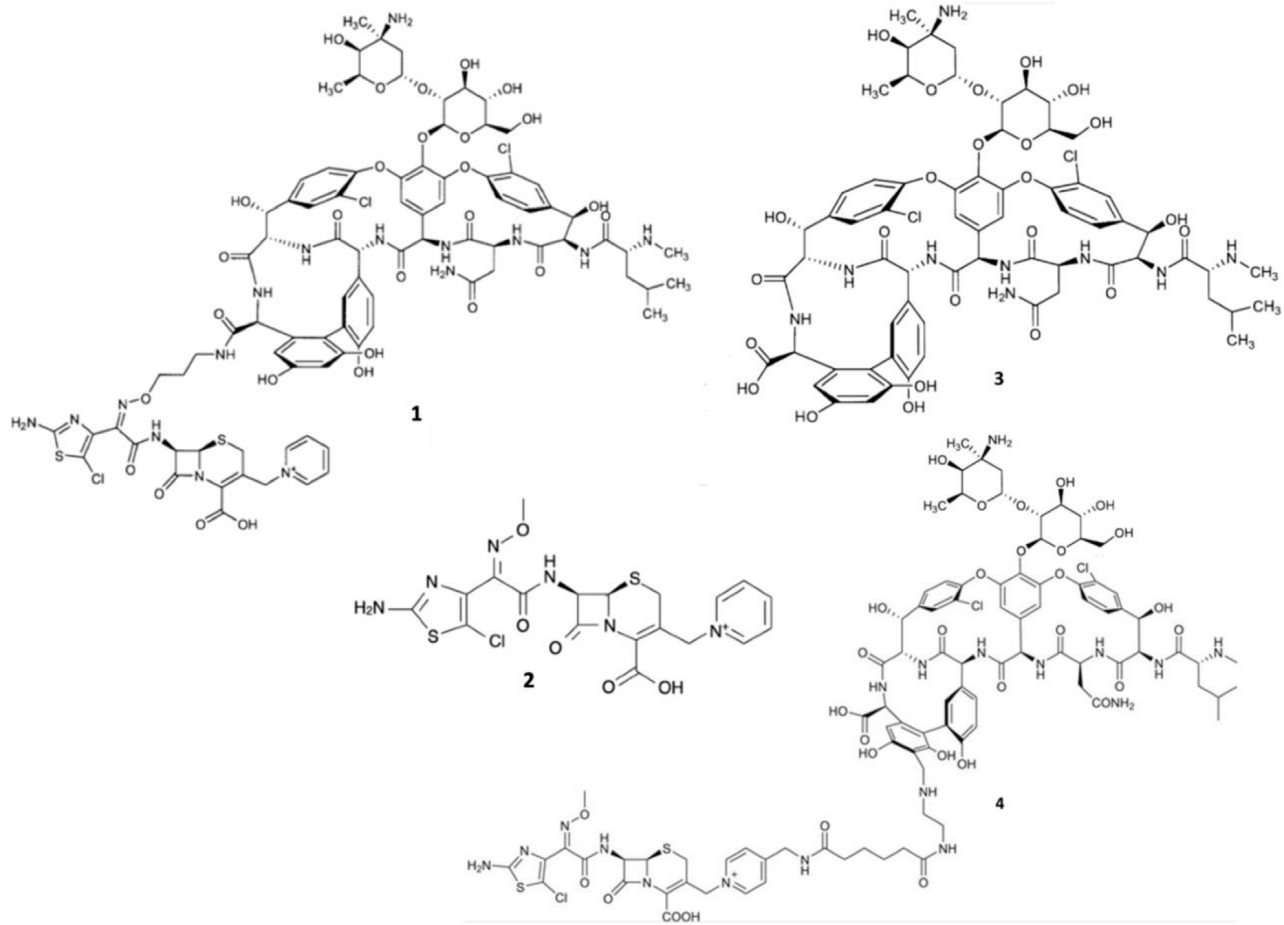
Structures of TD-1792 1, THRX-169797 2, vancomycin 3, and TD-1607 4.

However, even glycopeptide efficacy has been increasingly undermined by the emergence of vancomycin-intermediate *S. aureus* (VISA), and vancomycin-resistant *Enterococcus* (VRE) strains expressing seven resistance operons (VanA, VanB, VanC, VanD, VanE, VanF, and VanG) responsible for producing modified lipid II (Jubeh et al., 2020). VISA exhibits intermediate resistance to vancomycin via the excessive production of lipid II to sequester the glycopeptides (Sujatha and Praharaj, 2012; Butler et al., 2014). Moreover, the majority of VRE strains exhibit the VanA-phenotype, expressing lipid II with D-Ala-D-Lac epitopes of which vancomycin has overwhelmingly reduced binding affinity (Butler et al., 2014; Blaskovich et al., 2018). Expression of D-Ala-D-Lac is made possible primarily via *vanA*-operon expression and capable of being transferred interspecies via conjugation, which has led to the emergence of vancomycin-resistant *S. aureus* (VRSA) strains (Courvalin, 2006; Périchon and Courvalin, 2009; Sujatha and Praharaj, 2012). Therefore, it was surmised that to avert resistance to both *β*-lactams and glycopeptides, conjugating their pharmacophores to exploit the close molecular proximity of PBPs and lipid II could enable augmented bactericidal potency. Thus, Long and co-workers synthesized two *β*-lactam-glycopeptide hybrids, TD-1792 1 and TD-1607 4, from the pharmacophores of vancomycin 3 and the 3^rd^ generation cephalosporin derivative THRX-169797 2 (Figure 2) (Long et al., 2008a; Long et al., 2008b).

Among the two vancomycin-cephalosporin hybrids, TD-1607 4, was discontinued after two phase 1 trials (NCT01791049, NCT01949103) possibly owing to poor tolerability, while TD-1792 1 cleared phase 2 trials for the treatment of Gram-positive complicated skin and soft-tissue infections (Butler and Paterson, 2020; NCT01791049, 2013; NCT01949103, 2013; Long et al., 2008b; Umscheid et al., 2011). Consistent with its *in vitro* potencies, TD-1792 exhibited strong antibacterial activity *in vivo* (≥1-log_10_CFU kill) against all Gram-positive strains from methicillin-susceptible *S. aureus*, methicillin-susceptible *S. epidermidis*, methicillin-resistant *S. epidermidis*, penicillin-susceptible *S. pneumoniae*, *S. pyrogenes*, MRSA and VISA present in the murine neutropenic thigh infection models investigated by Stryjewski and co-workers (Stryjewski et al., 2012a). TD-1792 is administered intravenously IV), possesses a half-life of 9–13-h, is moderately bound to plasma proteins (∼50%) in humans, and is primarily excreted via renal filtration. Compared to vancomycin, fewer adverse effects (pruritus, headaches, gastrointestinal disorders, etc) were reported in the TD-1792 treatment group in the phase two safety and efficacy study carried out by Stryjewski and co-workers (Stryjewski et al., 2012b). Ergo, TD-1792 is reported to have successfully progressed to phase three trials for Gram-positive complicated skin and soft tissue infections (adisinsight, 2022; Butler and Paterson, 2020).

**FIGURE 3:**
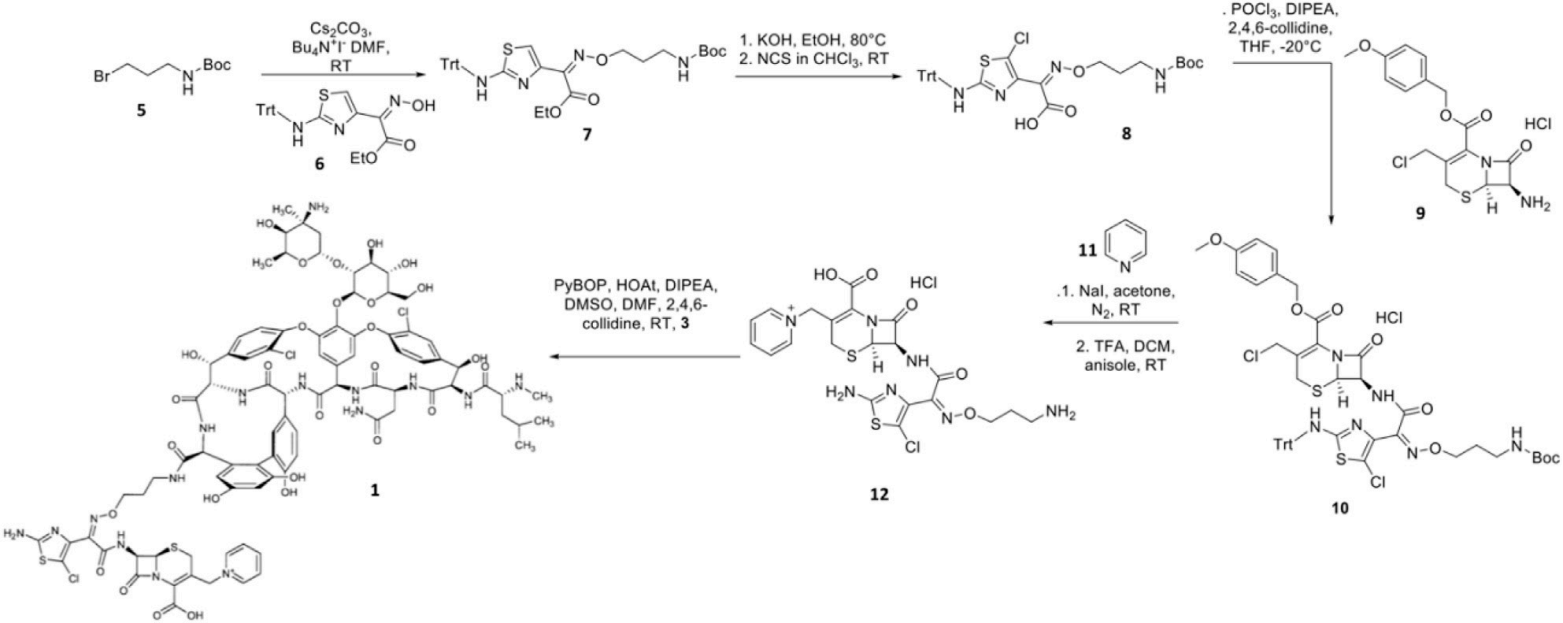
Synthetic scheme of TD-1792.

Synthesis of TD-1792 1 (Figure 3) was commenced by coupling N-Boc–protected bromopropylamine 5 with the trityl-protected aminothiazolyl residue 6 to yield intermediate **7**, which was then subjected to ester hydrolysis and chlorination with N-chlorosuccinimide (NCS), synthesizing Intermediate 8. Next, 8 was conjugated with 7-amino-3-chloromethyl-3-cephem-4-carboxylic acid p-methoxybenzyl ester 9. The resulting intermediate 10 was coupled with pyridine 11 and deprotected with trifluoroacetic acid (TFA) to yield THRX-169797 derivative 12. Finally, vancomycin 3 was conjugated directly to 12 through amide linkage to produce 1 (Long et al., 2008a). Cefilavancin effectively disrupts late-stage peptidoglycan synthesis via the inhibition of PBP and binding of lipid II D-Ala-D-Ala epitope, preventing peptidoglycan elongation and cross-linking concurrently (Figure 10A). Compared to penicillin G, cefilavancin exhibits substantial stability to staphylococcal *β*-lactamases congruent with that of THRX-169797 and is significantly unaffected by coexisting resistance mechanisms against multiple *β*-lactams (i.e., penicillin, methicillin, and oxacillin) and other antibiotic classes like fluoroquinolones (Blais et al., 2012; Stryjewski et al., 2012b). However, cefilavancin was primarily ineffective against Gram-negative pathogens just like vancomycin, owing to the impermeability of the outer membrane (Zhanel et al., 2008; Tyrrell et al., 2012).

### 3.2 Rifamycin hybrid candidates

**FIGURE 4:**
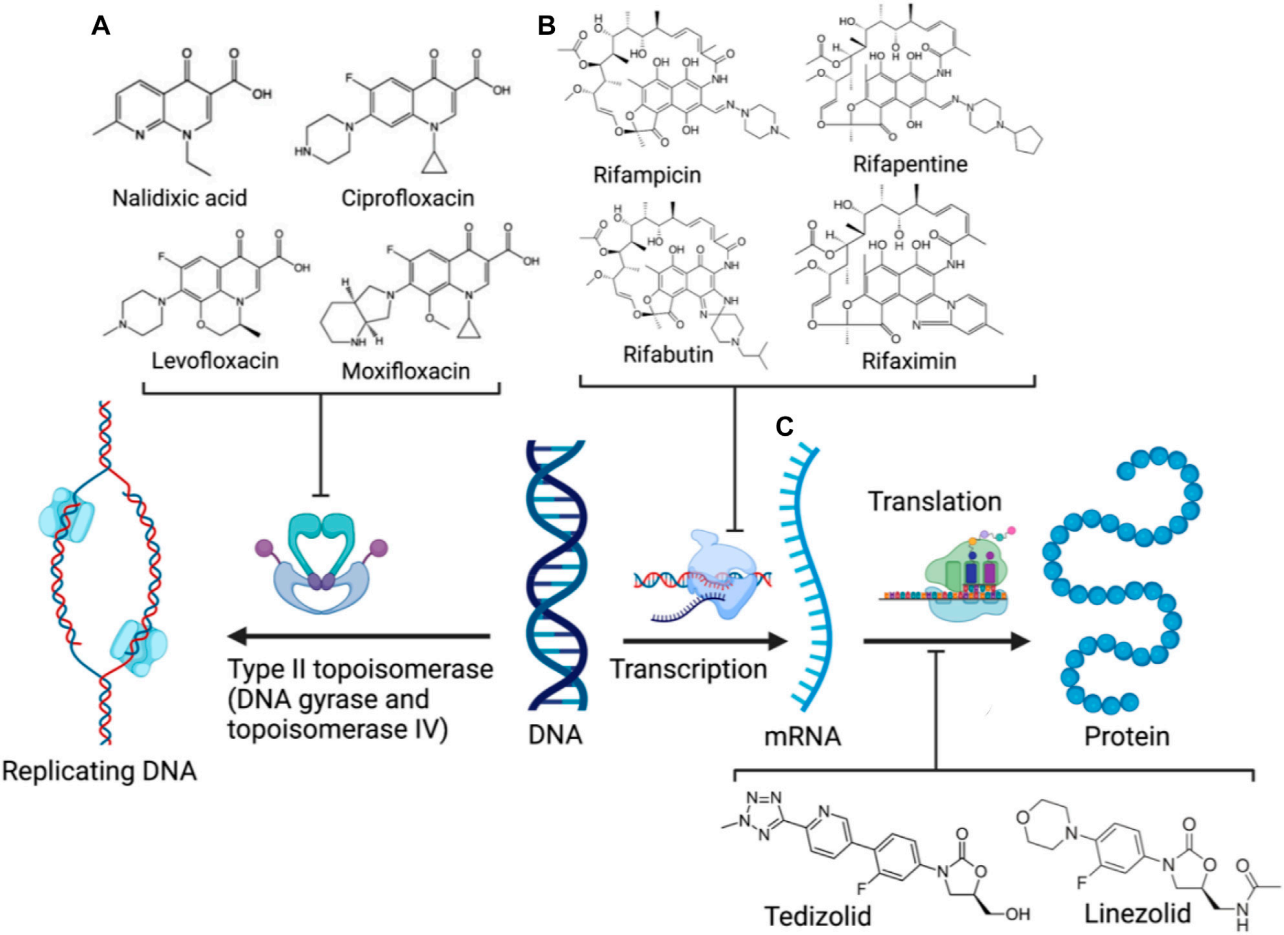
DNA replication and expression in bacteria and the corresponding clinical antibiotic classes, quinolones **(A)**, rifamycins **(B)**, and oxazolidinones **(C)** targeting the respective enzymes involved (Created with BioRender.com) (Bozdogan and Appelbaum, 2004; Goldstein, 2014; Pham et al., 2019).

Crucial bacterial processes also exploited by antibiotics include chromosomal replication and gene expression. As bacteria have small-sized circular genomes, DNA replication and transcription occur in proximity (Proshkin et al., 1979; Merrikh et al., 2012). Hence, the neighbouring enzymes involved in DNA replication and expression can be readily exploited by the relevant antibiotic classes/families in combination or tethered together. The rifamycin family (Figure 4B) is a potent antibiotic class employed against biofilm pathogens, functioning as DNA-dependent RNA polymerase (RNAP) inhibitors (Ma et al., 2007). However, resistance against the rifamycin developed rapidly as point mutations resulted in the production of low-affinity bacterial RNA polymerases (Goldstein, 2014), requiring the use of rifamycin in combination therapy to potentiate antibacterial activity (Ma and Lynch, 2016; Fisher et al., 2020). Quinolones (and their fluorinated derivatives, fluoroquinolones, Figure 4A) are broad-spectrum bactericidal antibiotics that inhibit the enzymes topoisomerase IV and DNA gyrase, arresting DNA replication and transcription (Pham et al., 2019). Nevertheless, significant resistance against quinolones evolved via chromosomal mutations, and the acquisition of plasmids carrying relevant resistance genes. The overexpression of efflux pumps, mutations in DNA replication enzymes, and enzymatic drug modification substantially diminish quinolone efficacy, likewise prompting combination therapy to mitigate resistance (Aldred et al., 2014). However, as some rifamycin/quinolone cocktails exhibit a degree of antagonism *in-vitro* and *in-vivo* (Neu, 1991; Murillo et al., 2008; Balasubramanian et al., 2012), it was postulated that the synthesis of rifamycin-quinolone hybrids could effectively overcome both rifamycin and quinolone resistance by preventing undesired drug-drug interactions (Fisher et al., 2020).

One rifampicin-fluoroquinolone hybrid, TNP-2092, passed phase two trials (NCT03964493) for the treatment of acute bacterial skin and skin structure infections (ABSSSI) and was designated as an orphan drug for treating prosthetic joint infections (PJIs), which are often associated with substantial morbidity rates and healthcare expenses (NCT03964493, 2019; Fisher et al., 2020; Tande and Patel, 2014). TNP-2092 can be administered either intravenously or orally (World Health Organization, 2021). When administered orally (PO), TNP-2092 was observed to accumulate in the gastrointestinal tract due to low absorption, resulting in reduced systemic exposure and bioavailability compared to IV administration, and is excreted in feces. Ergo, TNP-2092 exhibits considerable potency in *C. difficile* murine infection models, capable of clearing infections at low doses (6.67 mg/kg). Moreover, TNP-2092 was observed to exert potent activity (MIC ≤ 0.12 μg/ml) against urease-producing bacteria, including *Helicobacter pylori* (*H. pylori*) and *Salmonella*, compared to rifaximin which is conventionally applied for these infections. The expedited clearance of urease-producing species considerably alleviates hepatic infections associated with liver cirrhosis. Hence, TNP-2092 is placed under clinical investigation for treating gastrointestinal and hepatic infections (Yuan et al., 2020).

**FIGURE 5:**
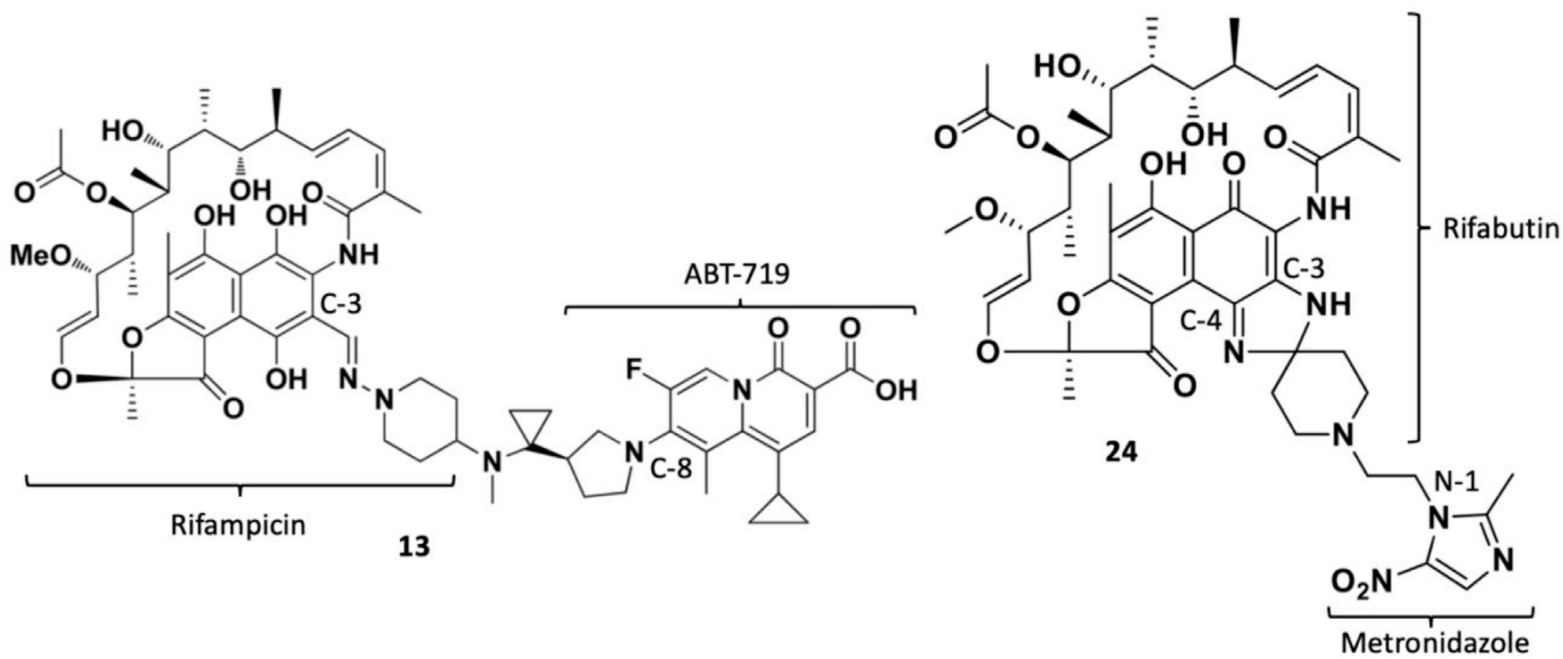
Structures of rifamycin hybrids TNP-2092 13 and TNP-2198 24.

**FIGURE 6:**
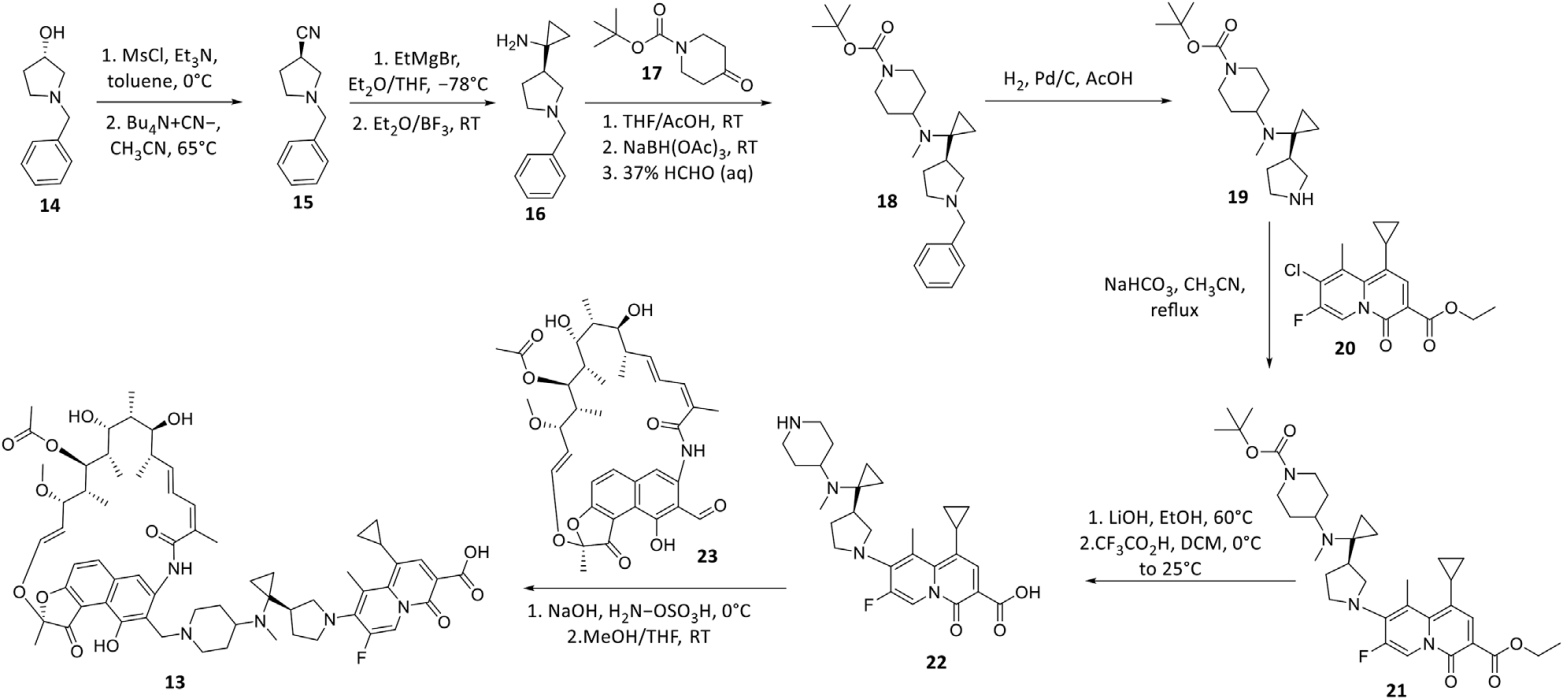
Synthetic scheme of TNP-2092.

TNP-2092 13 was designed via tethering the C-3 and C-8 side chains of rifampicin and the ciprofloxacin derivative ABT-719, respectively (Figure 5) (Ding et al., 2007; Ma and Lynch, 2016). Large-scale synthesis of 13 (Figure 6) commenced with the five-step synthesis of the linker component 19 from the precursor (S)-1-benzyl-3-hydroxypyrrolidine 14. Secondly, the linker 19 was conjugated with the ciprofloxacin derivative 20 under reflux in acetonitrile, producing 21, which was subjected to ester hydrolysis and deprotection with TFA, yielding 22. Finally, 22 is conjugated with the rifampin derivative 3-formalrifamycin 23 by converting the piperidine amino group of 22 into a hydrazine moiety, that is coupled with the C-3 aldehyde moiety of 23 to yield 13 (Ding et al., 2007). Biochemical characterizations of TNP-2092 indicated simultaneous inhibitory binding of RNAP and the type II topoisomerases, DNA gyrase, and DNA topoisomerase IV (Figure 10B), including common quinolone-resistant variants of the latter, accounting for potentiated bactericidal activity (Robertson et al., 2008). This enhanced activity of TNP-2092 was also confirmed by susceptibility tests involving a panel of *S. aureus* isolates carrying rifamycin or quinolone resistance alleles (or both), from which potency was observed (MIC ≤ 2 μg ml^−1^) against resistant isolates with either or both types of resistance alleles relative to rifampicin and ciprofloxacin, *per se* and in combination (MICs ≥8 μg ml^−1^) (Ma and Lynch, 2016; Fisher et al., 2020). Moreover, TNP-2092 is invulnerable to expulsion by fluoroquinolone efflux pumps, likely because of steric interference from the rifamycin pharmacophore, further verifying its low propensity for resistance development (Ma and Lynch, 2016). However, TNP-2092 shares a similar antibacterial spectrum of coverage as rifampicin, being less effective against most Gram-negative species owing to the limited permeability of the outer membrane to rifamycins (Yuan et al., 2020).

**FIGURE 7:**
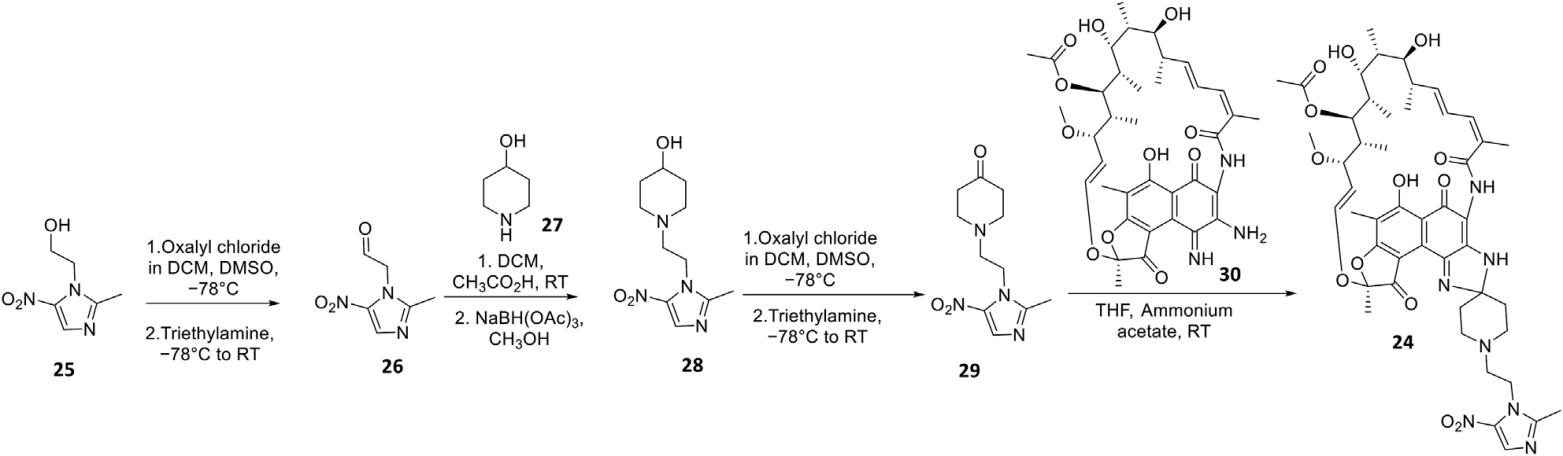
Synthetic scheme of TNP-2198.

Apart from fluoroquinolones, rifamycins were also hybridized with nitroimidazoles to overcome rifamycin resistance (Ma et al., 2022). Nitroimidazoles (E.g., metronidazole 25**)** are broad-spectrum antimicrobials utilized to treat anaerobic bacterial (Gram-positive and -negative), protozoal, and parasitic infections by yielding free radicals that inflict oxidative damage to bacterial DNA and other proteins (Mital, 2009; Ang et al., 2017). The rifamycin-nitroimidazole hybrid, TNP-2198, was produced by Ma and co-workers via tethering the C-3/C-4 and N-1 side chains of rifabutin and metronidazole, respectively, via an ethylene linker (Figure 5). Ma and co-workers synthesized TNP-2198 24 in milligram to kilogram quantities via a synthetic scheme (Figure 7) commencing from metronidazole 25. Firstly, 25 was subjected to Swern oxidation (using oxalyl chloride dissolved in DMSO, followed by quenching with triethylamine) to yield 26. Next, 26 was condensed with 4-hydroxypiperidine 27 and reduced using sodium triacetoxyborohydride in methanol to yield intermediate 28, which is used to produce intermediate 29 via Swern oxidation. Lastly, the ketone moiety of 29 is coupled with the C-3/C-4 amine and imine moieties of 3-amino-4-deoxy-4-imino-rifamycin S 30 to produce TNP-2198 24 (Omura and Swern, 1978; Ma et al., 2022).

TNP-2198 was observed to exert 4-fold greater potency than metronidazole against rifamycin-resistant *H. pylori* (MIC 0.5 μg ml^−1^ and 2 μg ml^−1,^ respectively) and 64–500-fold potent activity against both lab-generated rifamycin- and ciprofloxacin-resistant *C. difficile* strains, suggesting the ability of the metronidazole scaffold to enhance the bioactivity of the adjacent rifabutin pharmacophore. The synergy between the rifabutin and metronidazole pharmacophores of TNP-2198 was verified via time-kill assays in which TNP-2198 displayed rapid bactericidal activity (≥ 3.0 log_10_ CFU) compared to either rifamycin, metronidazole monotherapy or 1:1 M rifamycin/metronidazole combination. As determined by X-ray crystallography, synergistic bactericidal mechanisms occur via the rifampicin pharmacophore occupying the RNAP RNA exit cleft and the metronidazole scaffold simultaneously binding to the DNA template strand in the RNAP active center cleft via hydrogen bonding. Furthermore, under microaerophilic/anaerobic conditions, nitroreductase-catalyzed reduction of the metronidazole nitro (-NO_2_) moiety yields additional toxic TNP-2198 free radicals forming fatal covalent cross-links with other bacterial nucleic acids and proteins (Figure 10C).

Similar to TNP-2092, TNP-2198 exhibits high tissue distribution in the stomach, large intestine, gums, and vaginal tissue. Consistent with its potent *in vitro* activity, TNP-2198 prolonged the survival rate of hamster *C. difficile* infection models (at 100% for 6 days) subjected to a low dosing regimen of 5 mg/kg compared to metronidazole monotherapy at 100 mg/kg from which 100% mortality was observed following 4 days. Moreover, TNP-2198 substantially reduced mean bacterial titers at low concentrations (5 mg/kg) to levels (∼2.8 log_10_ CFU) comparable to that of the potent anti-*H. pylori* macrolide clarithromycin (10 mg/kg) in *H. pylori* murine infection models (Ma et al., 2022). Nitroimidazoles are known for exhibiting cytotoxicity and genotoxicity, warranting their development as prodrugs for administration (Nepali et al., 2019). Promisingly, TNP-2198 exhibits lower distribution in the central nervous system, which eliminates neurotoxicity associated with the metronidazole pharmacophore, possibly due to the hybrid’s larger molecular weight preventing access through the blood-brain barrier and the rifabutin pharmacophore enabling better bacterial targeting (Hartmann et al., 1967; Koike et al., 2020; Ma et al., 2022). Hence, TNP-2198 has since entered phase two trials for treating vaginosis and gastrointestinal infections caused by *H. pylori* and *C. difficile* (World Health Organization, 2021).

### 3.3 Oxazolidinone hybrid candidates

**FIGURE 8:**
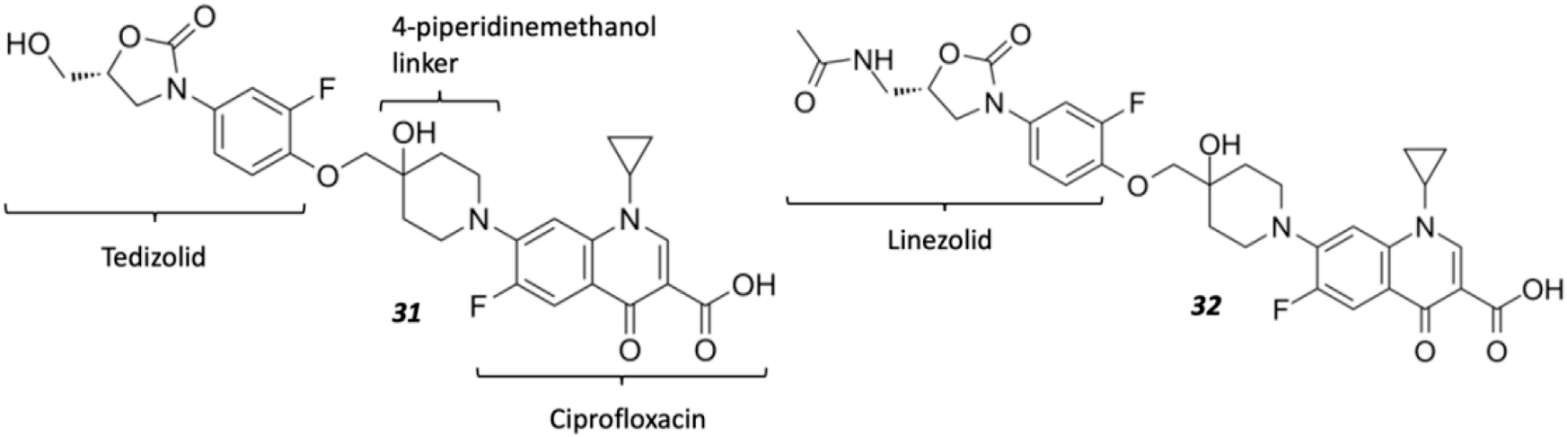
Structures of oxazolidinone hybrids Cadazolid 31 and DNV-3681 32.

Oxazolidinones (Figure 4C) are synthetic antibiotics derived from 2-oxazolidone, which occupy the P-site of the 50 S ribosomal subunit, inhibiting the assembly of the translation initiation complex and the translocation of peptidyl-tRNA from the A-site to the P-site, impeding mRNA translation (Bozdogan and Appelbaum, 2004). However, extensive resistance against oxazolidinones occurs via target modification (i.e., 23 S rRNA mutations in the 50 S subunit) and efflux (Brenciani et al., 2022). Several research groups have synthesized several oxazolidinone-fluoroquinolone hybrids, which were investigated for their potential to overcome oxazolidinone resistance and expand the spectrum to include Gram-negative pathogens, considering the ability of fluoroquinolones to permeate the outer membrane (Diver, 1989; Gordeev et al., 2003; Hubschwerlen et al., 2003). The oxazolidinone-fluoroquinolone hybrids with a 4-piperidinemethanol-derived linker were noted to display augmented potency and a reduced propensity for resistance development (Liu et al., 2019). Presently two such hybrids, cadazolid and DNV-3681 (Figure 8), cleared phase one trials (World Health Organization, 2021).

Cadazolid, a tedizolid-ciprofloxacin hybrid, was investigated as an alternative antibiotic to vancomycin and metronidazole for treating *C. difficile*-associated diarrhea (CDAD) (Locher et al., 2014). Inhibition observed from DNA topoisomerase assays was minimal compared to fluoroquinolones moxifloxacin and ciprofloxacin, suggesting cadazolid is primarily a protein synthesis inhibitor with weak inhibition of DNA synthesis as a secondary effect. This was affirmed via cryo-electron microscopy analysis, indicating that cadazolid occupies the ribosomal P-site via its oxazolidinone pharmacophore, which consequently projects the fluoroquinolone pharmacophore into the A-site, sterically preventing the elongation of the peptide chain by blocking the next aminoacyl-tRNA from occupying the A-site of the ribosomal translation complex (Scaiola et al., 2019). Cadazolid is administered orally and accumulates in the colon due to its poor aqueous solubility, resulting in low plasma concentrations and its primary excretion in feces (Baldoni et al., 2014). However, after two identically designed phase three trials (NCT01983683, NCT01987895) involving the treatment of CDAD (Gerding et al., 2019), further clinical developments were reported to be discontinued owing to its failure to achieve non-inferiority to vancomycin in one of the two trials (Theuretzbacher, 2020; fiercebiotech, 2018; Butler and Paterson, 2020).

**FIGURE 9:**
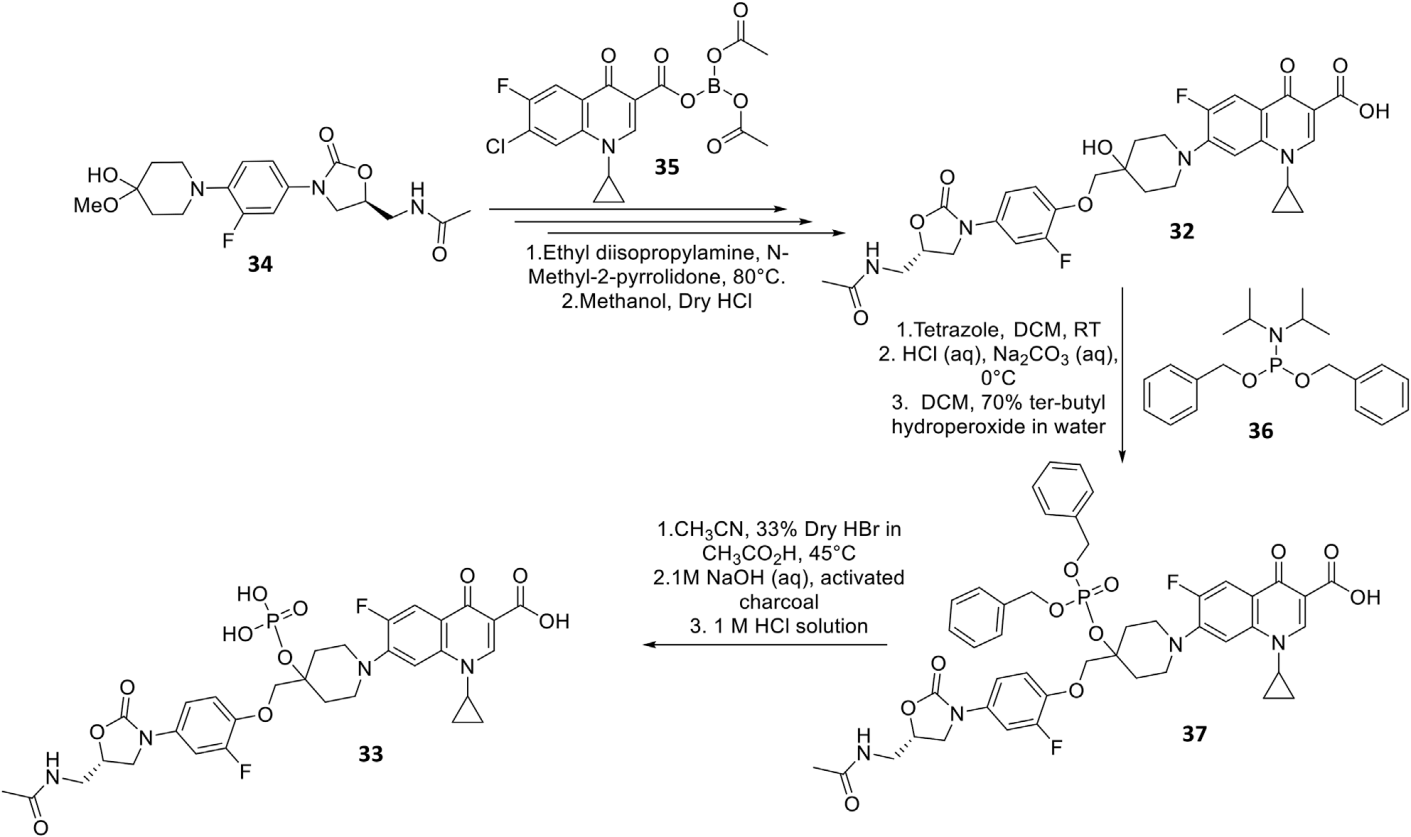
Synthetic scheme of DNV-3681 and DNV-3837 (Hubschwerlen et al., 2012).

**FIGURE 10:**
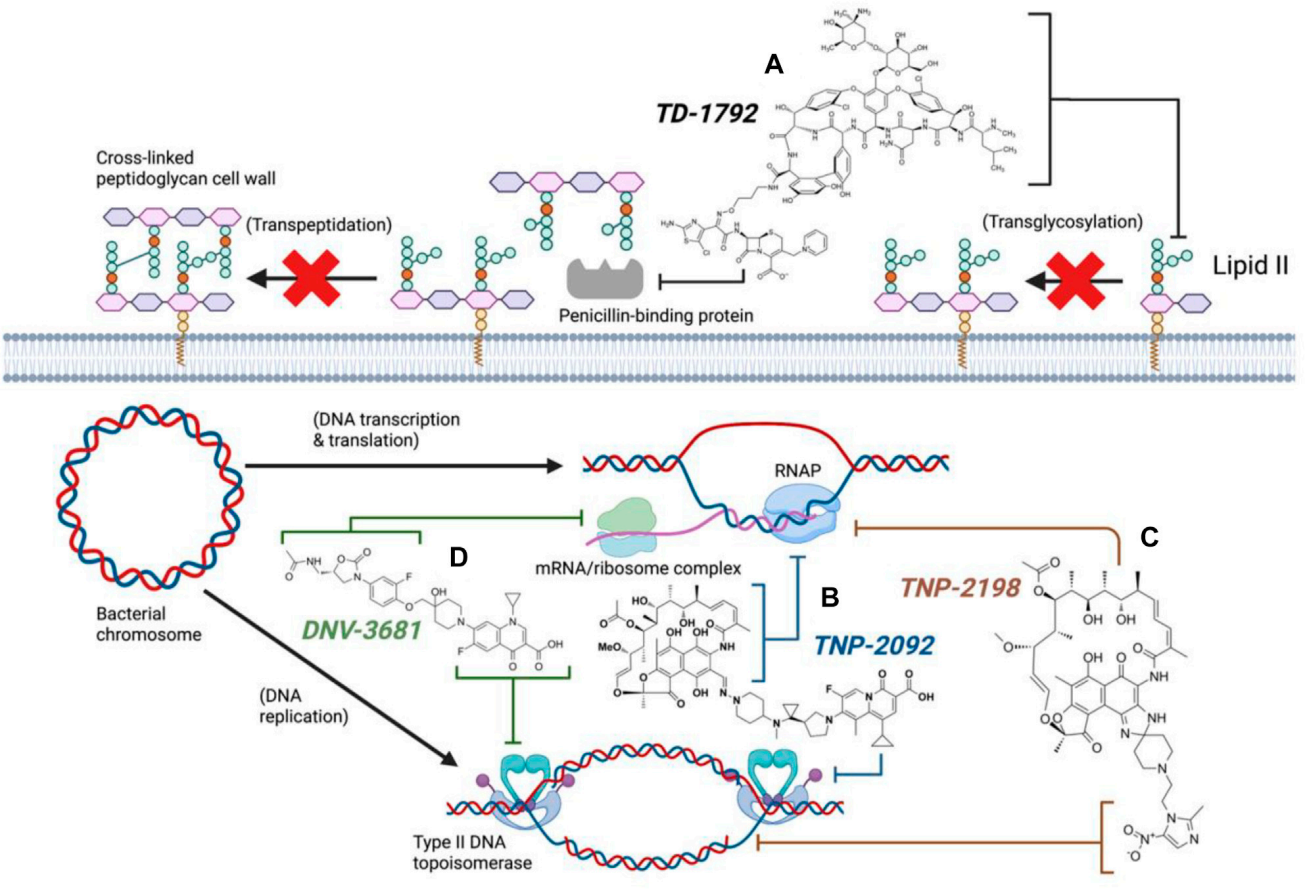
Antibacterial mechanisms of dual hybrid candidates (Created with BioRender.com). TD-1792 simultaneously binds lipid II and PBP (**A**, Black), TNP-2092 inhibits both RNA polymerase and type II DNA topoisomerases (**B**, Blue), TNP-2198 inhibits transcription and concurrently damages DNA (**C**, Brown), DNV-3681 inhibits both protein synthesis and DNA replication (**D**, Green).

DNV-3681 32 is another oxazolidinone-fluoroquinolone hybrid, comprised of linezolid and ciprofloxacin. It can either be administered PO, or IV as a water-soluble prodrug DNV-3837 33 (Figure 9) [NO_PRINTED_FORM]Promisingly, DNV-3681 exhibited 4-8-fold greater potency (MIC_50/90_ 0.032 μg ml^−1^/0.064 μg ml^−1^) against 114 *C. difficile* isolates compared to cadazolid (MIC_50/90_ 0.125 μg ml^−1^/0.5 μg ml^−1^) in susceptibility tests conducted by Rashid and co-workers, suggesting a comparatively stronger inhibition of DNA replication and transcription than cadazolid (Figure 10D) (Rashid et al., 2014). Thus, DNV-3681 effectively overcomes dual quinolone-oxazolidinone-resistant *C. difficile* isolates and exhibits superior efficacy compared to standard antibiotics for treating CDAD, including vancomycin, metronidazole, and ciprofloxacin (DNV3837, 2022; Freeman et al., 2017). In a phase one investigation conducted by Dalhoff and co-workers involving twelve healthy male volunteers, DNV-3681 displayed minimal systemic absorption and was generally well tolerated following IV administration. DNV-3681 is distributed readily in the colon, where it exhibits potent activity against Gram-positive organisms (e.g., *Clostridia*, *Enterococci*, and *S. aureus*) whilst leaving normal Gram-negative microflora generally unscathed (Dalhoff et al., 2015). Currently, DNV-3681 progressed to phase two trials (NCT03988855) for the treatment of CDAD (Kullar et al., 2020).

## 4 Future directions

The concept of dual-acting hybrid antibiotics currently holds significant promise in overcoming bacterial resistance since some compounds have progressed to phase three (E.g., cadazolid and cefilavancin). However, this novel approach is not without challenges. For instance, the complexity of designing chemical synthetic procedures may affect overall yields and potentially impair the intrinsic activity of the hybrids synthesized (Ma and Lynch, 2016; Domalaon et al., 2018; Lungu et al., 2022). Other obstacles involve the drug permeability impediments in Gram-negative bacteria due to increased molecular weight (> 600 Da), as well as the meticulous work needed to understand the mode of action and determine the benefits of the hybrid compounds over conventional antibiotics, such as the possibility of delaying the evolution of resistance (Pokrovskaya et al., 2009; Shavit et al., 2017; Koh Jing Jie et al., 2022). To remedy some of the challenges, the antibiotic hybridization strategy has expanded to include conjugation to adjuvants - small molecules or biologics without intrinsic antibacterial activity but potentiate antibiotic activity through anti-resistance mechanisms, bypassing membrane barriers or immune cell stimulation (Wright, 2016; Melander and Melander, 2017; Koh Jing Jie et al., 2022). To illustrate, siderophore adjuvants have been conjugated with antibiotics (E.g., cefiderocol, Fetroja^®^) to successfully bypass the outer membrane barrier of Gram-negative bacteria by exploiting the iron-uptake pathway (Fetroja FDA, 2022). With improvements in synthetic procedures, conjugation of adjuvants with the current dual hybrid candidates may assist in overcoming membrane permeability issues. Despite the challenges, the antibiotic hybrid strategy remains a viable approach to expanding the antimicrobial arsenal and minimizing the severe attrition experienced in the antibiotic discovery pipeline. As more data could be unraveled in the future, this field could be broadened and thereby, there will be a great optimism that human ingenuity will pave the way for the next generations of hybrids that can equip the antibiotic arsenal.
